# Prevalence and Determinants of Home Delivery among Women with Easy Access to Health Facilities in Sub‑Saharan African Countries: A Multi‑level Mixed Effect Analysis

**DOI:** 10.5334/aogh.4615

**Published:** 2025-01-24

**Authors:** Berhan Tekeba, Alebachew Ferede Zegeye, Deresse Abebe Gebrehana, Tadesse Tarik Tamir

**Affiliations:** 1 Department of Pediatrics and Child Health Nursing, School of Nursing, College of Medicine and Health Sciences, University of Gondar, Gondar, Ethiopia; 2 Department of Medical Nursing, School of Nursing, College of Medicine and Health Sciences, University of Gondar, Gondar, Ethiopia; 3 Department of Internal Medicine, School of Medicine, College of Medicine and Health Sciences, University of Gondar, Gondar, Ethiopia

**Keywords:** Home delivery, No distance difficulty, Health facility, DHS sub‑Saharan Africa

## Abstract

*Introduction:* Most maternal deaths are associated with home deliveries, which account for half of births in low‑income countries. To develop appropriate policies and methods that could aid in addressing the issues, it is important to understand the burden of home delivery despite having easy access to health facilities in low‑income nations such as sub‑Saharan Africa. In addition, identifying and prioritizing determinants could help executives to review their perinatal policies. Therefore, this study aimed at assessing the prevalence and factors associated with home delivery among women who have access to health facilities.

*Methods:* A population‑based cross‑sectional study was done. The most recent Demographic and Health Survey (DHS) data from 22 sub‑Saharan African (SSA) countries from 2015 to 2022 were used. A total weighted samples of 493,396 women who gave birth at home despite having access to health facilities were included in the study. The data were examined using Stata 17. A multi‑level logistic regression model was used to identify factors associated with lactation–home delivery despite easy access to health facility. The adjusted odds ratio at the 95% confidence interval (Cl) was computed to assess the strength and significance of the association between explanatory and outcome variables. Factors with a *p*‑value of < 0.05 are declared statistically significant.

*Results:* The pooled prevalence of home delivery among women who had easy access to health facilities in 22 SSA countries was 23.67% (95% CI, 23.55–23.79). After adjusting for confounders, being above 35 years, being a rural resident, being from the poorest and lowest wealth quintile, and living in rural regions all increase the likelihood of home birth among women in sub‑Saharan Africa who have easy access to healthcare. Women with higher education, women who have optimal antenatal care (ANC) visits, women involved in healthcare decisions in households, and households with health insurance coverage reduce the odds of home delivery among women who have easy access to health facilities in sub‑Saharan Africa.

*Conclusion:* According to this study, a higher portion of women in sub‑Saharan Africa who had easy access to medical facilities gave birth at home. The study’s findings demonstrated that factors at the individual and community levels influence home delivery with easy access to health facilities. Policymakers, the government, health planners, and implementers must therefore understand the burden of the problem and should increase media coverage, enhance health insurance coverage, empower women, and mobilize resources for maternal care.

## Introduction

Home delivery refers to having a baby in an unclean, non‑clinical setting [1, 2]. It is a critical site for the majority of maternal deaths occur from obstetric complications, including hemorrhage, sepsis, hypertension, embolisms, and other causes of death [3]. In industrialized nations, experienced healthcare professionals assist with almost 99% of deliveries, whereas in poor nations, this number is only 62% [4]. Even though the number of maternal deaths worldwide has recently decreased, low‑ and middle‑income nations are still unable to meet the minimally acceptable rates [5]. This is especially high in sub‑Saharan African (SSA) countries, where high birth rates, high lifetime risks of maternal mortality, weak health systems, poor health‑seeking behaviors, and poverty have resulted in decades of stagnation in the rate of reduction in maternal mortality [6, 7].

It has been discovered that utilizing facility delivery with a trained attendant is an effective strategy to provide a noticeable improvement in the health outcomes for mothers and newborns [8]. Access to healthcare is a measure of human well‑being that is constrained by numerous geographically varying factors [9, 10]. The most pressing of these is the length of time and distance that an individual must travel to a health facility that is both appropriately staffed and equipped, especially in rural areas where travel expenses and times are frequently higher [11]. Poor transportation infrastructure and a lack of motorized transport can make the problem worse. As a result, persons who have difficulty accessing medical facilities are less likely to seek care because home delivery has been normalized, which increases maternal morbidity and death [12].

According to previous literature findings, there are many factors associated with home delivery in SSA countries. Accordingly, maternal age, antenatal care (ANC) visit, distance to a health facility, media exposure, residence, parity, type of facility, number of children ever born, marital status, cohabitation period, wealth index, birth order, knowledge of obstetric complications, birth preparedness, and complication readiness plan significantly determine home delivery [1, 13–18].

The World Health Organization advises that every delivery be supervised by a trained birth attendant who can recognize and address any difficulties or provide basic treatment and referrals [19]. Nonetheless, the proportion of deliveries by skilled birth attendant (SBAs) remains below the acceptable levels. Skilled attendants assist with approximately half of all deliveries in sub‑Saharan Africa. Even in countries where antenatal care (ANC) is practiced, a considerable number of births occur at home [20, 21].

The Safe Motherhood Initiative (SMI) formerly launched at a conference held in Nairobi, Kenya [19], and Sustainable Development Goal 3 (SDG‑3) strive to reduce maternal mortality to the minimum acceptable tolerance rate. Nevertheless, despite this goal, the maternal and newborn mortality rates decline at a slower pace, particularly in low‑income sub‑Saharan African countries. Evidence showed that, although skilled birth attendants can save the lives of women, only 59% of births were held at health facilities between 2012 and 2017 in sub‑Saharan Africa [22]. Thus, more investigation is needed to determine what else could cause home delivery and related consequences if access to a health facility is not impeded by distance.

It is obvious that home delivery is higher in SSA countries, with higher negative consequences for both the mother and the newborn. Despite this fact, women in SSA countries still deliver their babies at home, which accounts for a significant proportion of maternal morbidity and mortality. Distance from a health facility is a known risk factor for home delivery, as prior research studies have shown [1, 20]. Nevertheless, despite having easy access to healthcare facilities, many women in sub‑Saharan Africa still give birth at home. However, the up‑to‑date magnitude of home delivery and its associated factors among women who had easy access to health facilities are not well studied in SSA countries. Therefore, this study aimed at assessing the magnitude of home births and its associated factors among women who have easy access to health facilities in sub‑Saharan African nations using the most recent Demographic and Health Survey (DHS) dataset that will help to update officials and may also help them prioritize barriers against home delivery despite having easy access to health facilities.

## Methods

### Data source

The most recent DHS data from the 2015–2022 Demographic and Health Survey in 22 sub‑Saharan African countries were used. The countries included in this study were Angola, Burkina Faso, Benin, Burundi, Cameroon, Ethiopia, Gabon, Ghana, Gambia, Guinea, Kenya, Liberia, Mali, Malawi, Nigeria, Rwanda, Sierra Leone, Tanzania, Uganda, Zanzibar, Zambia, and Zimbabwe. The survey was used to gather information on a number of demographic characteristics related to the health of women and children, including the place of delivery [23]. The details of the methodology employed in the DHS were documented by Corsi and Neuman [24]. Total weighted samples of 493,396 women who gave birth at home and had access to healthcare services from 22 [22] SSA countries were included in our study. The datasets for DHS are available at http://dhsprogram.com/data/available-datasets.com.

### Source and study population

All reproductive‑aged women who deliver at home despite having access to health facilities in SSA countries were the source population. The study populations were all reproductive‑aged women in SSA countries who deliver at home despite having access to health facilities in selected enumeration areas (EAs).

### Sample size and sampling procedure

In general, all selected national surveys used the most recent census frame. DHS samples are often classified by geographic region (provinces) and, within each region, by urban or rural areas. The majority of DHS sample designs are multi‑stage sampling techniques based on existing census frames. Enumeration areas (EAs) were the primary sampling units, and households (HH) were the secondary sampling units. Following the listing of households, equal probability sampling was used to select a specified number of households in the designated clusters. Weighted values were computed using the individual kid’s record (KR) file in the DHS dataset to restore the representativeness of the sample data. Finally, this study comprised a total of 493,396 women from 22 SSA countries who delivered at home despite having easy access to health facilities. A total of 47 countries are found in sub‑Saharan Africa. Of these countries, only 41 had DHS reports. From these, after excluding countries that had no recent DHS since 2015 and countries where the DHS dataset was not publicly available, 22 countries were included in the study.

## Variables of the Study

### Outcome variable

The outcome variable of this study was home delivery among women with easy access to health facilities. It was derived from the response given on the DHS questionnaire to “distance to a health facility” and “women’s place of delivery.” Women who responded “no” to the distance difficulty question were considered to have easy access to health facilities and those who responded “yes,” to have difficulty accessing health facilities. Then, we excluded women who had delivered in health facilities. By keeping women who delivered at home in Stata, we categorized them using “yes = 1” for those who underwent home delivery despite easy access to health facilities and “no = 0” for those who delivered at home who had difficulty accessing health facilities.

### Independent/Explanatory variables

Both individual‑ and community‑level variables were included in the study.

### Individual‑Level factors

Maternal age and education level, the mother’s work status, paternal education level, the household head’s sex, wealth index, media exposure, the mother’s involvement in decision‑making about healthcare, health insurance availability, duration of pregnancy, the mother’s not going alone, the women’s permission to go to a health facility, the obtaining of money to go to a health facility, the ANC visit, multiple pregnancies, and birth interval history of terminated pregnancies were individual‑level variables.

### Community‑Level factors

Place of residence, the community’s ANC use, the community’s poverty level, the community's media exposure, the community’s illiteracy level, and region were community‑level variables.

### Operational definition

“Difficulty accessing a health facility” refers to a woman living more than 1 hour from a health facility by local means of transportation, or the availability of a health facility within a travel distance of 5 kilometers [25].

### Statistical analysis

We used Stata version 17. Software was used to enter, code, clean, record, and analyze the data. Stata was initially developed by the Computing Resource Center in California [26].

### Model building

We have applied weighting to restore representativeness and get a better statistical estimate. In DHS, data variables are nested by clusters, and those within the same cluster show more similarities than those with separate clusters. Thus, using the traditional logistic regression model violates the assumptions of independent observation and equal variance across clusters. Therefore, a more advanced model is required to account for cluster characteristics. A multi‑level, multivariable logistic regression model was employed in the study to identify the variables associated with home delivery among women with easy access to health facilities. In the analysis, four models were fitted. The first model (the null model) was fitted only to the outcome variable to assess the variability of the status of home delivery among women who had easy access to health facilities between clusters or to assess the intra‑correlation coefficient. The second model was fitted to individual‑level factors only; the third model was fitted to community‑level factors only; and the final model (model 4) was fitted to both individual‑ and community‑level variables.

### Random effect (measure of association)

Random effect analysis, proportional change in variance (PCV), median odds ratios (MOR), and intra‑cluster correlation coefficients (ICC) were used to evaluate the community‑level variability of home delivery among women who had easy access to health facilities. Deviance was utilized to confirm model fitness, and model 4 has been determined to be the best‑fit model due to its lowest deviance.

### Fixed effect (measure of association) analysis

A bivariate analysis was done to identify the factors eligible for multivariable analysis; in bivariate analysis, a variable with a *p*‑value of less than 0.2 was included for multivariable analysis. In the multivariable analysis, variables with a *p*‑value of less than 0.05 were deemed to be significant predictors of home delivery among women who had access to a health facility. The adjusted odds ratio (AOR) along with its 95% confidence interval (CI) was used to declare the level of significance.

### Ethical statement and consent to participate

The Demographic and Health Survey (DHS) data from 22 sub‑Saharan African countries were gathered in compliance with national and international ethical standards. Ethical clearance was provided by the Institutional Review Board of ICF International and the United States Centers for Disease Control and Prevention. At the time of data collection for the Demographic and Health Survey, all study participants were asked for informed oral consent, and data were collected anonymously. Data collectors explained the purpose of the study and explained that participation in the study was voluntary. Privacy and confidentiality were ensured by following the ethical requirements of the research. The study involved minimal risk for study participants. More details regarding DHS data and ethical standards are available online at http://www.dhsprogram.com.

## Result

**Figure 1:**
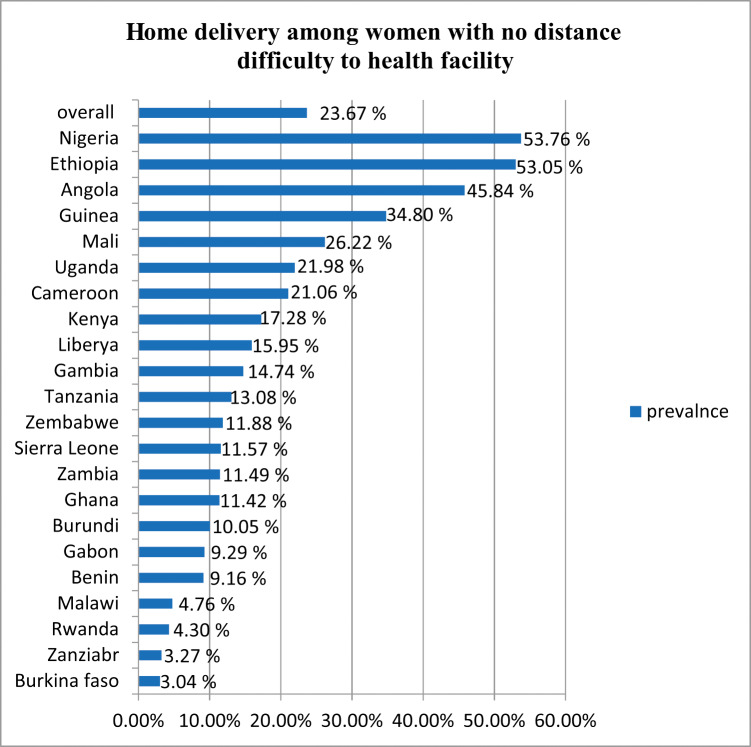
Prevalence of home delivery among women who had no distance difficulty when it came to accessing health facilities in 22 sub‑Saharan African countries.

The study was conducted among 22 [22] sub‑Saharan African countries using the most recent DHS data from the 2015–2022 period. The pooled prevalence of home delivery among women who had easy access to health facilities in 22 SSA countries was 23.67% (95% CI, 23.55–23.79). The lowest prevalence was observed in Burkina Faso (3.04%), and the highest prevalence was observed in Nigeria (53.76%; Figure 1).

### Sociodemographic and health‑facility‑related factors of home delivery

A total weighted sample of 493,396 women who gave birth at home who had access to healthcare facilities was included in the study. More than two‑thirds (70.84%) of respondents were aged 20–34 years. Over one‑third (35.00%) of those surveyed were employed. More than one‑third (38.03% and 37.06%) of respondents and their husbands, respectively, had no formal education. More than three‑fourths (78.8%) of households were headed by men. More than one‑third (42.03%) of respondents were involved in healthcare decision‑making. The majority (96.34%) of births were singletons. The majority (85.01%) of respondents had no health insurance. Half (50.68%) of respondents had no exposure to media. More than half (59.09%) of respondents resided in rural areas. The majority (92.91%) of respondents had no permission to go to a health facility. Nearly two‑thirds (62.15%) of women had not gotten money for treatment. Nearly half (48.39%) of respondents resided in a community with access to media. Half (49.29%) of the respondents were also from communities with high poverty levels (Table 1).

**Table 1:** Sociodemographic and health facility variables of home delivery.

PARAMETER	RESPONSE	FREQUENCY	PERCENT (%)
Maternal age (years)	15–19 20–34 ≥ 35	27,758 349,908 116,27	5.6 70.84 23.54
Maternal education	No education Primary Secondary Higher	187,859 139,768 138,706 27,607	38.03 28.3 28.08 5.59
Maternal working status	Yes No	172,864 321,076	35.00 65.00
Paternal education	No education Primary Secondary Higher	159,160 108,375 117,096 44,888	37.06 25.23 27.26 10.45
HH sex	Male Female	388,796 104,600	78.8 21.2
Wealth index	Poorest Poor Middle Rich Richest	96,410 96,656 100,545 100,114 100,004	19.54 19.59 20.36 20.27 20.25
ANC visit	Non‑optimal Optimal	133,019 225,965	37.05 62.95
Involved in healthcare decisions	Yes No	180,662 249,164	42.03 57.97
Duration of pregnancy	Preterm Term Post‑term	19,273 419,595 14,486	4.25 92.55 3.2
Birth interval	< 24 months 24–48 months ≥ 48 months	71,093 209,606 97,916	18.78 55.36 25.86
History of terminated pregnancy	Yes No	426,758 67,182	13.6 86.40
Multiple pregnancies	Yes No	18,093 475,847	3.66 96.34
Health insurance	Yes No	66,302 375,977	14.99 85.01
Media exposure	Yes No	243,625 250,312	49.32 50.68
Get money for treatment	Yes No	173,761 285,339	37.85 62.15
Wanted to go alone to a health facility	Yes No	22,494 436,606	4.9 95.1
Got permission to go to a health facility	Yes No	32,566 426,534	7.09 92.91
**Community‑level factors**			
Place of residence	Urban Rural	202,056 291,884	40.91 59.09
Community’s ANC use	High Low	294,611 199,329	59.65 40.35
Community’s media exposure	High Low	239,032 254,908	48.39 51.61
Community’s illiteracy rate	High Low	208,388 285,552	42.19 57.81
Community’s poverty level	High Low	243,483 250,457	49.29 50.71
Region	Central West East South	64,608 247,170 179,071 3,091	13.08 50.04 36.25 0.63

### Random effect and model fit statics

The random effect estimates were observed by fitting four models (null model, model I, model II, and model III). The null models showed that there was a significant variation in the likelihood of home delivery within sub‑Saharan African countries (σ2 = 1.86, *p* < 0.001). The ICC in the null model revealed that 36.12% of the total variation in home delivery among women with easy access to health facilities was due to the difference between clusters. Moreover, the MOR of 3.65 in the null model implies that the odds of home delivery among mothers who had easy access to health facilities were 3.55 times higher in the high cluster than in the lower cluster when clusters were randomly selected. The proportional change in variance (PCV) in the final model revealed that 17.2% of the variation in home delivery among women with easy access to health facilities was explained by both individual‑level and community‑level variables. The final model (model III) is considered the best‑fit model because it has the lowest deviance (Table 2).

**Table 2:** Random effect analysis and model comparison.

PARAMETER	NULL MODEL	MODEL I	MODEL II	MODEL III
**Variance**	1.86	1.78	1.35	1.54
**ICC**	36.12%	35.11%	29.09%	31.88%
**MOR**	3.65	3.55	3.02	3.25
**PCV**	Reference	4.3%	27.42%	17.20%
**LLR**	−5,560.19	−4,249.086	−3,264.49	−2,899.39
**Deviance**	11,120.38	8,498.17	6,528.98	5,798.78

ICC = Intracluster Correlation Cofficent, MOR = Median Odds Ratio, PCV = Proportional Change in Variance, LLR = Loglikilhood Ratio.

### Factors associated with home delivery among women who had no difficulty accessing health facilities

A multi‑level multivariable logistic regression analysis was done to identify factors significantly associated with home delivery among women with easy access to health facilities. Accordingly, maternal age, maternal education level, wealth index, ANC visit, involvement in healthcare decisions, access to health insurance, access to media exposure, and place of residence were significantly associated with home delivery among women with no distance difficulty when it came to accessing health facilities.

Being in the extreme maternal age range above 35 years increases the odds of home delivery by 15% (AOR = 1.15; 95% CI, 1.12–1.18) as compared with the maternal age range of 20–34 years. Mothers with higher education had 63% lower odds of an AOR = 0.37 (95% CI, 0.33–0.41) when it came to home delivery as compared with women with no formal education. Women in the poorest households had six times (AOR = 6.04; 95% CI, 5.72–6.37) and women in poor households had four times (AOR = 4.63; 95% CI, 4.40–4.88) higher odds of home delivery, respectively, as compared with women in the richest wealth quintile. Women with optimal ANC visits had 62% decreased odds of home delivery (AOR = 0.38; 95% CI, 0.37–0.39) as compared with women with non‑optimal ANC visits. Women involved in the healthcare decisions in their households had 11% decreased odds of home delivery (AOR = 0.89; 95% CI, 0.87–0.91) as compared with women not involved in the healthcare decisions in their household. Households having health insurance coverage decrease the odds of home delivery by 54% (AOR = 0.46; 95% CI, 0.44–0.49) as compared with households not having health insurance. Women who reside in rural areas have 42% higher odds of home delivery (AOR = 1.42; 95% CI, 1.37–1.47) as compared with women who reside in urban areas (Table 3).

**Table 3:** Factors associated with home delivery among women with no difficulty accessing health facilities in sub‑Saharan African countries.

PARAMETER	RESPONSE	MODEL I AOR (95% CI)	MODEL II AOR (95% CI)	MODEL III AOR (95% CI)
Maternal age (years)	15–19 20–34 ≥ 35	1.28 (1.16–1.42) 1 1.20 (1.17–1.23)		1.05 (0.95–1.16) 1 1.15 (1.12–1.18)*
Maternal education	No education Primary Secondary Higher	1 0.72 (0.70–1.74) 0.63 (0.60–1.65) 0.37 (0.33–0.41)		1 0.72 (0.69–1.74) 0.58 (0.56–1.61) 0.37 (0.33–0.41)*
Maternal working status	Yes No	1 1.45 (0.91–1.49)		1 1.47 (0.95–1.51)
Paternal education	No education Primary Secondary Higher	1 0.96 (0.93–1.01) 1.31 (0.95–1.17) 1.55 (0.96–1.65)		1 0.98 (0.94–1.01) 1.11 (0.97–1.16) 1.58 (0.98–1.68)
HH sex	Male Female	1 0.86 (0.83–1.1)		1 0.86 (0.83–1.10)
Wealth index	Poorest Poor Middle Rich Richest	5.62 (5.34–5.92) 4.84 (4.60–5.09) 0.35 (0.3–1.37) 0.22 (0.21–1.23) 1		6.04 (5.72–6.37)* 4.63 (4.40–4.88)* 0.3 (0.31–1.39) 0.21 (0.21–1.26) 1
ANC visit	Optimal Non‑optimal	0.41 (0.40–0.42) 1		0.38 (0.37–0.39)* 1
Involved in healthcare decisions	Yes No	0.95 (0.93–0.98) 1		0.89 (0.87–0.91)* 1
Duration of pregnancy	Preterm Term Post‑term	0.49 (0.45–1.3) 1 0.64 (0.60–1.69)		0.53 (0.49‑1.8) 1 0.58 (0.5–1.56)
Birth interval	< 24 months 24–48 months ≥ 48 months	1 0.76 (0.74–1.79) 0.52 (0.50–1.54)		1 0.77 (0.75–1.80) 0.52 (0.50–1.54)
History of terminated pregnancy	Yes No	1.06 (0.92–1.09) 1		1.05 (0.91–1.08) 1
Multiple pregnancies	Yes No	1 0.77 (0.71–1.84)		1 0.76 (0.70–1.83)
Health insurance	Yes No	0.42 (0.40–0.44) 1		0.46 (0.44–0.49)* 1
Media exposure	Yes No	1 1.06 (1.02–1.09)		1 1.43 (1.40–1.47)*
Get money for treatment	Yes No	1 1.27 (0.94‑1.30)		1 1.22 (0.8‑1.25)
Wanted to go alone to a health facility	Yes No	1 0.82 (0.78–1.86)		1 0.78 (0.74–1.83)
Getting permission to go	Yes No	1 0.76 (0.72–1.79)		1 0.88 (0.84–1.92)
**Community‑level factors**				
Place of residence	Urban Rural		1 3.61 (3.54–3.69)	1 1.42 (1.37–1.47)
Community’s ANC use	High Low		2.44 (0.13–2.79) 1	0.39 (0.33–1.45) 1
Community’s media exposure	High Low		1.58 (0.36–1.84) 1	0.92 (0.77–1.09) 1
Community’s illiteracy rate	High Low		0.66 (0.57–1.77) 1	0.80 (0.68–1.94) 1
Community’s poverty level	High Low		0.88 (0.77–1.01) 1	1.13 (0.96–1.33) 1
Region	South Central West East		1 1.6 (0.7–1.4) 1.3 (0.85–2.6) 1.3 (0.74–1.27)	1 2.0 (0.24–2.62) 0.82 (0.46–1.71) 0.13 (0.09–1.74)

*statisticaly significant (*p*‑value <0.05).

## Discussion

This study found that the prevalence of home delivery among women who had easy access to health facilities in SSA countries was 23.67% (95% CI, 23.55–23.79). The lowest prevalence was observed in Burkina Faso (3.04%), and the highest prevalence was observed in Nigeria (53.76%). This study is in line with the study done in East Africa [17]. However, the eastern Africa study does not exclude women who had easy access to health facilities, which may further lower the existing prevalence. This study is lower than other studies done in sub‑Saharan Africa [27]. The reason for the discrepancy could be that the data used in the earlier study only covered the years 2010–2018. Consequently, our analysis reflects the most recent timeframe during which countries may have made progress toward SDG‑3 by reducing maternal mortality by facilitating facility delivery. This study finding was also lower than the global prevalence (low‑ and middle‑income countries) of home births, which was 28% (95% CI, 0.24–0.33). The possible explanation for this could be that the global prevalence was observed among all women who deliver at home, meaning that both women who had and those who did not have easy access when it came to accessing institutional delivery were incorporated, and the highest prevalence was observed. However, our study only incorporated home delivery among women who had easy access to health facilities, which may lower the prevalence.

This study found significant factors associated with home delivery among women who had easy access to health facilities in sub‑Saharan African countries. Accordingly, maternal age, maternal education level, wealth index, ANC visit, involvement in healthcare decisions, access to health insurance, access to media exposure, and place of residence were significantly associated with home delivery among women with no difficulty accessing health facilities.

Women with higher education had a lower probability of delivering at home as compared with uneducated women. This is supported by the studies done in different parts of the world [28–32]. This can be explained by the increased likelihood of improved female self‑determination, positive attitudes, and financial independence that come with higher levels of maternal education [33, 34]. Moreover, educated women are more likely to recognize problems during pregnancy and childbirth and to seek high‑quality care. Consequently, compared with moms who lack education, they are more likely to utilize maternal healthcare [35]. Thus, educated moms are more likely to seek maternal health services, including health facility delivery.

According to this study, older mothers had higher odds of home delivery as compared with women of optimal reproductive age. This is supported by other studies [21]. This could be because older mothers who have given birth more than younger ones may perceive they are more experienced at giving birth, which means that they may perceive delivery anywhere, including at home, as not problematic for them. Furthermore, older mothers tended to believe that giving birth at home was less risky as that had been their previous experience. In addition, older women use more traditional approaches than younger mothers do [36].

Women in rural areas had higher odds of home delivery as compared with their urban counterparts. This is also supported by other studies [18, 37]. This is because rural residents in sub‑Saharan African countries have limited access to healthcare facilities, little awareness of institutional delivery services, and a scarcity in terms of institutional delivery services [38]. Additionally, women might be obliged to give birth at home due to a shortage of qualified medical professionals, longer travel times, carelessness, and extremely restricted access to obstetric care for rural residents.

Women who had no exposure to media had higher odds of home delivery as compared with their counterparts [39]. A possible reason for the increase in home delivery among women who are not exposed to media is that, as they miss out on exposure to mass media, which offers high awareness and knowledge about pregnancy, the promotion of institutional delivery, information regarding birth‑related complications, and changes in women’s attitudes, social norms, and behaviors that may lead to high access to health facility delivery are also missed out on, and they end up with home delivery [40]. The role of media in pregnant women’s choice of birth location necessitates improved media availability. Furthermore, health information conveyed through the media has an important influence on determining women’s health‑seeking behavior. They are more aware of the advantages of continued maternal healthcare utilization, warning indicators, and pregnancy‑related problems than their counterparts [41, 42].

Women having an optimal ANC visit decreases the likelihood of home delivery as compared with women having no optimal ANC visit. This is supported by other studies’ findings [43, 44]. This is because women may be able to obtain the best ANC follow‑up guidance from medical professionals regarding where to give birth and get information about risks of and problems with home delivery through focused prenatal care obtained during an ANC visit. In addition, women who received the best ANC visit have an opportunity to receive early detection and treatment of pregnancy‑related danger signs, allowing them to give birth in a medical institution [45].

Women being involved in healthcare decision‑making reduces the likelihood of home delivery as compared with their not being involved. This is supported by other findings [46, 47]. Since women are typically the primary caretakers, better results will be achieved when they have the ability to make important decisions about their health [48]. Choosing the place of delivery requires decision‑making by mothers and their close relatives [49–51]. Furthermore, independent women had greater access to and advocacy for medical facilities during childbirth [52]. Therefore, one acknowledged strategy for boosting maternal healthcare utilization is to improve the status of women’s empowerment in poor nations [53].

Households having health insurance coverage decreases the odds of home delivery as compared with households not having health insurance coverage [54, 55]. Compared with women who paid out of pocket, those with health insurance reported fewer delays in their decision to seek care, go to a hospital, and receive care there [56]. Pregnant women who have health insurance are more likely to choose to give birth in a medical facility [57]. Therefore, to lower maternal and infant mortality, health insurance should be advocated for and implemented in every nation on the planet.

We also found that women in the poorest wealth quintile had higher odds of delivering at home compared with those in the highest wealth quintile. This is in line with other studies [32, 58, 59]. It is obvious that wealthier women are more likely to deliver in a health facility than their poor counterparts [18, 42, 60]. This may be the result of financial hardships that impoverished women face, such as the expense of transportation and purchasing additional supplies when giving birth in a medical institution [31]. Furthermore, because institutional delivery entails fees related to pregnancy and childbirth, women from lower socioeconomic backgrounds are more likely to choose home delivery [15, 61].

## Conclusion

The study found that a higher proportion of women who had easy access to health facility delivery delivered at home. Factors at the individual and community levels were significantly associated with home delivery. The main factors that determined home delivery despite having access to health facilities in our study were the mother’s age and education level, residence in a rural area, access to media, health insurance coverage, optimal ANC visits, the mother’s involvement in healthcare decision‑making, and wealth index. Policymakers, the government, health planners, and implementers must therefore understand the burden of the problem, increase media coverage, enhance health insurance coverage, empower women, and mobilize resources for maternal care.

### Strength and limitation

The strength of this study stemmed from the inclusion of 22 nations with varying social, cultural, and economic contexts. Because the study is based on data from a nationwide survey, it may help programmers and policymakers create effective national initiatives. The cross‑sectional nature of the study design precluded the use of the results of this investigation in drawing conclusions about the causal relationship between the independent factors and the outcome. Recall bias may also exist because DHS is a cross‑sectional survey that relies on self‑reporting by respondents.

## Figures and Tables

**Table:** 

ANC	Antenatal care
EA	Enumeration area
ICC	Inter‑cluster correlation coefficient
LR	Logistic regression
MOR	Median odds ratio
SBA	Skilled birth attendant
SDG	Sustainable Development Goal
SSA	Sub‑Saharan African
PCV	Proportional change in variance

## Data Availability

The most recent data from the Demographic and Health Survey were used in this study, and it is publicly available online at http://www.dhsprogram.com. The datasets used and/or analyzed during the current study are available from the corresponding author on reasonable request.
